# Tractography in Type 2 Diabetes Mellitus With Subjective Memory Complaints: A Diffusion Tensor Imaging Study

**DOI:** 10.3389/fnins.2021.800420

**Published:** 2022-04-06

**Authors:** Jun Wang, Laiyang Ma, Guangyao Liu, Wenjuan Bai, Kai Ai, Pengfei Zhang, Wanjun Hu, Jing Zhang

**Affiliations:** ^1^Department of Magnetic Resonance, Lanzhou University Second Hospital, Lanzhou, China; ^2^Department of Endocrine, Lanzhou University Second Hospital, Lanzhou, China; ^3^Department of Clinical Science, Philips Healthcare, Xi’an, China

**Keywords:** type 2 diabetes, white matter, cognitive function, memory, diffusion tensor imaging

## Abstract

The brain white matter (WM) structural injury caused by type 2 diabetes mellitus (T2DM) has been linked to cognitive impairment. However, the focus was mainly on the mild cognitive impairment (MCI) stage in most previous studies, with little attention made to subjective memory complaints (SMC). The main purpose of the current study was to investigate the characteristics of WM injury in T2DM patients and its correlation with SMC symptoms. In a group of 66 participants (33 HC and 33 T2DM-S), pointwise differences along WM tracts were identified using the automated fiber quantification (AFQ) approach. Then we investigated the utility of DTI properties along major WM tracts as features to distinguish patients with T2DM-S from HC *via* the support vector machine (SVM). Based on AFQ analysis, 10 primary fiber tracts that represent the subtle alterations of WM in T2DM-S were identified. Lower fractional anisotropy (FA) in the right SLF tract (*r* = −0.538, *p* = 0.0013), higher radial diffusivity (RD) in the thalamic radiation (TR) tract (*r* = 0.433, *p* = 0.012), and higher mean diffusivity (MD) in the right inferior fronto-occipital fasciculus (IFOF) tract (*r* = 0.385, *p* = 0.0029) were significantly associated with a long period of disease. Decreased axial diffusivity (AD) in the left arcuate was associated with HbA_1c_ (r = −0.368, *p* = 0.049). In addition, we found a significant negative correlation between delayed recall and abnormal MD in the left corticospinal tract (*r* = −0.546, *p* = 0.001). The FA of the right SLF tracts and bilateral arcuate can be used to differentiate the T2DM-S and the HC at a high accuracy up to 88.45 and 87.8%, respectively. In conclusion, WM microstructure injury in T2DM may be associated with SMC, and these abnormalities identified by DTI can be used as an effective biomarker.

## Introduction

Type 2 diabetes mellitus (T2DM) is a long-term systemic metabolic disease characterized by insulin resistance and hyperglycemia, accounting for up to 90% of all diabetes cases (Quattrocchi et al., 2020). T2DM-related complications can also cause injury to the central nervous system (CNS) in addition to the retina, kidneys, and peripheral nerves. There is increasing evidence that T2DM significantly increases the risk of Alzheimer’s disease, dementia, and vascular dementia (Cukierman et al., 2005; Yaffe et al., 2012; Bordier et al., 2014; Degen et al., 2016). Approximately 11.3% of elderly patients with T2DM have suffered from cognitive impairment (Bruce et al., 2003). Cognitive impairment in T2DM usually manifests as functional deficits in working memory, attention, and execution (Zhang et al., 2014; Srikanth et al., 2020). Subjective memory complaints (SMC) refers to subjective experiences of cognitive function decline which is a kind of self-reported abnormality (Hong et al., 2019). Compared with middle-aged people without diabetes, the proportion of T2DM patients with SMC has increased by 19% (Biessels and Despa, 2018; Fiore et al., 2019). SMC can incite anxiety in patients, leading to a poorer quality of life and increased healthcare utilization (Waldorff et al., 2009). The etiology and clinical significance of SMCs is unclear, but these complaints are associated with objective cognitive decline or with depression, anxiety, and psychosocial stressors (Vale et al., 2012). SMC has been reported to exhibit biological and physiological brain changes similar to those seen in Alzheimer’s disease. Whether SMC is attributable to brain structure changes in diabetic patients and cognitive dysfunction in the future is still controversial (Biessels and Despa, 2018; Bruce et al., 2019; Kawagoe et al., 2019).

Neuroimaging has been used to non-invasively characterize the changes of brain structure in patients with T2DM, mainly showing a decrease in gray matter volume and loss of axonal integrity (Li et al., 2020). Diffusion tensor imaging (DTI) is an inspection method that can effectively observe and track brain white matter (WM) fiber bundles, detect microstructural abnormalities, and assess the integrity of WM bundles in the brain (Reijmer et al., 2013; Vaeggemose et al., 2020). Compared with traditional structural MRI, DTI seems to be a more sensitive biomarker of cognitive decline caused by aging and axial diffusivity (AD; Schiavone et al., 2009; Zhuang et al., 2012). Relevant studies have shown that differential brain structural abnormalities between T2DM and healthy control (HC) may reflect pathological mechanisms underlying the effect of blood glucose fluctuation to the brain (Moulton et al., 2015; Alotaibi et al., 2021). Abnormalities in specific WM tracts can lead to disruption in information transfer and interruption of pathways within important brain regions (Xie et al., 2017; Sundar et al., 2019). The limited sample size, the variability of demographic characteristics of the patients, and the diversity of methodological methodologies may all contribute to the inconsistency of different studies. In a study using tract-based spatial statistics (TBSS), DTI-derived indexes were abnormal in several brain regions of T2DM, such as the right corpus cingulum (Xiong et al., 2016). Previous studies have provided some information on the impact of mild cognitive impairment (MCI) in T2DM (Sun et al., 2018; Xiong et al., 2020). A large amount of evidence has shown that T2DM with MCI will be an injury to the structure of the brain. The main damaged fiber tracts include the corpus callosum (Liang et al., 2019), the bilateral front limbs of the internal capsule (Zhuo et al., 2019), the bilateral posterior thalamus radiation (Tan et al., 2016), etc. The T2DM with amnestic MCI showed decreased fractional anisotropy (FA) in the right inferior fronto-occipital fasciculus (IFOF) and the right inferior longitudinal fasciculus (ILF; Gao et al., 2019).

In the past, most T2DM-related DTI studies have surveyed whole-brain WM diffusion metrics using either voxel-based analyses (VBA). It is inevitably affected by the size of smoothing kernel. Although TBSS (Sanjari Moghaddam et al., 2019) technology was used later, the influence of cross fibers on the results was still not solved. Meanwhile, many studies are limited to basic DTI-derived indexes such as FA and mean diffusivity (MD), in which the tracts were defined by a region of interest (ROI) approach (Hoogenboom et al., 2014; Cui et al., 2020). Automated fiber quantification (AFQ; Yeatman et al., 2012) is a widely used and successful tractography method in detecting structural abnormalities of the WM pathway, which enables us to segment the fiber tracts and identify the abnormal points accurately and automatically, avoiding the influence of smooth kernel (Sarica et al., 2017; Dou et al., 2020). An support vector machine (SVM) model is a classical machine learning algorithm, which can help us better distinguish the specific WM injury patterns of T2DM with SMC (T2DM-S) and find effective image biomarkers (Zhou et al., 2018). During the occurrence and development of the disease, the destruction of fiber tracts follows certain objective rules. A large number of studies have been done on healthy elderly patients and T2DM; even so, the impact of subtle cognitive impairment on T2DM has not been fully elucidated at the early stage of effective intervention. The main purpose of the current study was to investigate the characteristics of WM injury in T2DM patients and its correlation with SMC symptoms. In the present study, we hypothesize that WM disruption may vary along fiber tracts in T2DM and may provide potential candidate hallmarks for the pathological state of disease. We attempt to explore whether T2DM was associated with SMCs and the feasibility of AFQ tractography combined with SVM for identification of disease biomarkers.

## Materials and Methods

### Participants

The study was approved by the Medical Ethics Committee of the Lanzhou University Second Hospital and conducted according to the principles expressed in the Declaration of Helsinki. Each participant was aware of the purpose and risk of the study and signed informed written consent forms.

Between April 2018 and December 2020, 66 subjects (right-handed) were prospectively recruited in this investigation, including 33 T2DM patients (with SMC symptoms, T2DM-S) [9 females, 24 males; mean age 56.3 years, standard deviation (SD) 7.80] from Lanzhou University Second Hospital outpatient or inpatient according to the diagnostic criteria of the World Health Organization standards (Alberti and Zimmet, 1998), and 33 age-, sex-, and education-matched HC [15 females, 18 males; mean age 52.47 years, standard deviation (SD) 6.96] were recruited from the community. SMCs were confirmed by an affirmative answer to both of the following questions: (1) “Are you complaining about your memory?” and (2) “Is it a regular complaint which lasts more than 6 months?” (Jessen et al., 2014; Teipel et al., 2017). Namely, there were no informant-based complaints of memory impairment or decline, and Mini-Mental State Examination (MMSE) score ≥27 (Folstein et al., 1975; Kawagoe et al., 2019). In addition, in view of the high incidence rate of T2DM in elderly people, it is difficult to distinguish SMC origin from T2DM itself and other diseases, such as Alzheimer’s disease, as elderly people are more likely to exhibit neurodegenerative diseases. Therefore, middle-aged subjects (≤65) patients were recruited in order to clearly demonstrate the pathology of cognitive impairment.

The exclusion criteria for participants in this study included the following items: (1) a history of mental, neurological disorders; (2) lacunar infarction and WM hyperintensity; (3) diabetic retinopathy or nephropathy; (4) diabetic peripheral neuropathic pain; (5) degenerative disorders, such as Parkinson’s disease, and (6) hypertension or hyperlipidemia.

### Neuropsychological and Laboratory Testing

All patients underwent complete neuropsychological assessments and laboratory tests, including MMSE, MoCA, blood biochemistry, lipids and cholesterol levels, plasma glucose and glycosylated HbA_1c_ levels. Detailed information about demographic and clinical characteristics are shown in Table 1 and Supplementary Table S1.

**TABLE 1:** Demographic and clinical characteristics for each group.

	T2DM-S	HC	t/χ^2^	*p*-Value
Age (year)	56.30 ± 7.80	52.47 ± 6.96	1.705	0.095^a^
Sex (female/male)	9/24	15/18	2.36	0.125^b^
Education (years)	11.09 ± 2.61	11.59 ± 3.02	0.576	0.569^c^
SBP (mmHg)	118.33 ± 9.84	119.24 ± 9.36	0.312	0.756^a^
DBP (mmHg)	77.39 ± 17.95	70.12 ± 9.51	1.873	0.067^c^
BMI	22.07 ± 1.53	21.94 ± 1.38	0.280	0.779^a^
MoCA	26.06 ± 2.00	27.53 ± 1.44	1.903	0.063^c^
Visuospatial/executive	4.39 ± 0.83	4.27 ± 0.84	0.591	0.932^c^
Attention	5.42 ± 0.66	5.46 ± 0.56	0.200	0.366^c^
Delayed recall	4.18 ± 1.46	4.06 ± 0.86	0.406	0.685^c^
MMSE	28.06 ± 2.60	29.06 ± 1.25	1.834	0.072^a^
FBG (mmol/L)	12.50 ± 3.40	5.11 ± 1.63	11.26	0.000*
HbA_1C_ (mmol/mol)	0.11 ± 0.03	N/A	N/A	N/A
Duration (y)	6.23 ± 5.01	N/A	N/A	N/A

*Mean ± standard deviation (SD) is reported.*

*T2DM-S, type 2 diabetes mellitus with subjective memory complaints (SMC) symptoms; HC, health controls; MMSE, mini–mental state examination; MoCA, montreal cognitive assessment; SBP, systolic blood pressure; DBP, diastolic blood pressure; FBG, fasting blood glucose; BMI, body mass index; N/A, means that there is no relevant data.*

*^a^Two-sample t-test, two tailed unless otherwise indicated.*

*^b^χ^2^ test.*

*^c^Two-sample t-test with Welch’s correction.*

*p < 0.05.

### Data Acquisition

MRI images were acquired with an eight-channel phased-array head coil 3.0T scanner (Siemens Verio, Erlangen, Germany). To limit machine noise and minimize head motion, earplugs and tight foam cushioning were utilized separately. Single-shot SE-EPI sequence was used in DTI. The scanning parameters were TR = 8,700 ms, TE = 90 ms, layer thickness = 3.0 mm, matrix = 128 × 128, flip angle = 90°, FOV = 230 mm × 230 mm, 64 gradient directions at *b* = 1,000 s/mm2, plus one *b* = 0 image. At the same time, 3D high-resolution magnetization prepared rapid acquisition with gradient echo (MPRAGE) sequence was used for sagittal thin-layer T1WI to provide anatomical information for the location of anterior-posterior commissure (AC-PC). The scanning parameters are as follows: TR = 1,900 ms, TE = 2.79 ms, slice thickness = 1.0 mm, matrix = 256 × 256, the FOV = 256 mm × 256 mm, and flip angle = 9°, and 196 volumes. The parameters of the subjects were consistent, and the quality control was carried out by a professional technical staff.

### Automated Fiber Quantification Procedure

Open-source software (MrDiffusion and AFQ)^1^ ^,2^ (Yeatman et al., 2012) was utilized primarily *via* Matlab (The MathWorks, Natick, MA, United States) platform. Effects from subject motion were attenuated using the six-parameter rigid-body realignment. We then generated cortical and WM surfaces and brain region parcellations using the Freesurfer program.^3^ These will be used for segmenting the major WM tracts following tractography. DtiInit was used to preprocess DTI image, which can run eddy/motion correction and co-register to anatomy and products dt6 files stored in standard directory structures used for AFQ pipeline. Meanwhile, the MRtrix3 package^4^ was used to control susceptibility distortions, Gibbs ringing, and bias field inhomogeneities.

After completing the preceding preprocessing steps, the whole-brain probability streamlines were tracked using MRtrix3’s tckgen with constrained spherical deconvolution (CSD) and anatomically constrained tractography (ACT). The step size is 1 mm, the angle threshold is 45°, the threshold value of fiber bundle direction distribution is 0.05, and the number of streamlines is set to 1 million (Smith et al., 2012). Then we put the dt6 structure into the AFQ processing flow together with the fiber bundles generated by MRtrix3 using the iFod algorithm. The fiber bundles are segmented according to the predefined template of 20 main fiber bundles in the brain. Two fiber tracts were discarded based on known inconsistencies in automatic identification (bilateral cingulum hippocampus). FA was then assessed along each fiber group centroid using spline interpolation. The contribution of a single fiber to the core estimate is weighted based on the likelihood that the fiber is a member of the given fiber tract, computed as the Mahalanobis distance. The mean FA was then computed for each fiber tract on an individual subject basis. The same method applies to mean diffusivity (MD), radial diffusivity (RD), and AD, and each fiber bundle is divided into 100 equidistant points.

### Machine Learning Analyses

Four kinds of pointwise diffusion indicators (FA, MD, RD, and AD) of each fiber tracts were used as raw features, including 7,200 for each participant. Multivariate imputation was applied between the variables using a regressor model, in which each feature with missing values is modeled as a function of other features. Then we utilized Z-score standardization to eliminate the impact of measurement units on the results. Two feature selection methods (two sample *t*-test and variance) were adopted in this study. The *p*-value of two sample *t*-test was 0.05. The threshold of variance was set to 1. Before machine learning, the two groups were labeled (T2DM: 0, HC: 1), mixed, and then divided into training and testing sets in a 7:3 ratio. We selected 46 cases as the training data set (30/16 = positive/negative) and another 20 cases as the independent testing data set (12/8 = positive/negative). Then SVM prediction models using optimal parameter set was trained using leave-one-out cross-validation (LOOCV) and predicted on the test data sets (Abraham et al., 2014). Here, we used the linear kernel SVM because it was easier to explain the coefficients of the features for the final model. The performance of the models was evaluated from the sensitivity, specificity, and area under the curve (AUC). All the above processes were implemented with scikit-learn machine learning library (v0.24.1) on Python (3.7.6). Scripts used for all analyses are available on a web page named AFQ-Browser^5^ (Yeatman et al., 2018).

### Statistical Analysis

SPSS statistics for Windows, Version 24.0, was used for all statistical analyses (IBM, Chicago, IL, United States). For demographic data, we used two sample *t*-test and Mann–Whitney *U*-test for continuous variable. Simultaneously, the gender difference was compared using the chi-square (χ^2^) test. We used partial correlation analyses and linear regression to study the correlation between DTI-derived indexes and various clinical variables, such as disease duration time, blood glucose level, glycosylated hemoglobin, blood lipid index, etc., controlled with age, sex, and years of education as potential covariates. The display form of figures depends on recently published articles (Chandio et al., 2020).

## Results

### Demographic and Clinical Characteristics

Demographic and cognitive information of T2DM-S and HC are listed in Table 1. There were no significant intergroup differences in age, sex, and education level between groups (*p* > 0.05).

### Group Differences of Automatic Fiber Quantification Approach

**FIGURE 1:**
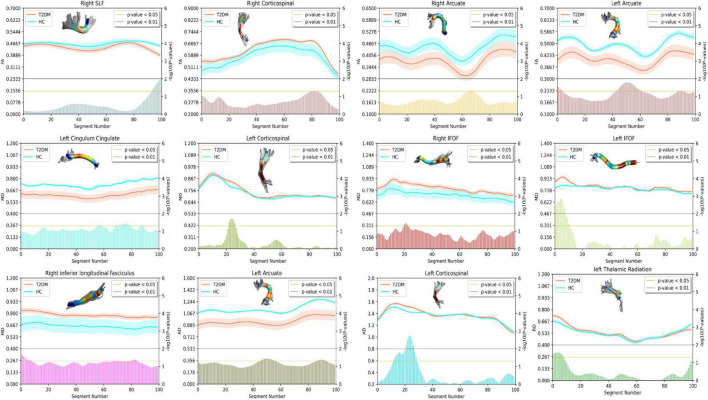
Plots summarizing group differences for diffusion tensor imaging (DTI)-derived indexes. Only fiber tracts with difference after point-wise comparison are shown. The *X*-axis represents the fiber segment, the left *Y*-axis represents the average DTI parameters, and the right *Y*-axis represents the negative logarithm of *p*-value. In the figure, the yellow horizontal line represents *p* < 0.05, and the gray horizontal line represents *p* < 0.01. The figure is plotted using seaborn (https://seaborn.pydata.org/) and AFQ’s function, as well as refer to the relevant contents in the DIPY toolkit (https://dipy.org).

According to the results of AFQ, 10 different fiber tracts exhibited significant alterations, including the bilateral corticospinal tracts (CST), the bilateral fronto-occipital fasciculus (IFOF), the bilateral arcuate, the right ILF, the left thalamic radiation (TR), the right superior longitudinal fasciculus (SLF), and the left cingulum cingulate (CC). Figure 1 shows the pointwise significant difference values of WM fiber tracts between T2DM-S and HC (Banfi et al., 2019)^6^. Table 2 shows the mean FA value of each fibers and group difference between the two groups. For along-tract results (shown as Table 3) (Colby et al., 2012)^7^, patients with T2DM-S had significantly reduced FA in the right SLF, bilateral arcuate. Increased MD values are shown in the left CST, left IFOF, right ILF, left CC, and right IFOF. We also find increased AD in left CST, reduced AD in left arcuate, and increased RD in the left TR relative to HC.

**TABLE 2:** Mean FA (× 100) of 18 fiber tracts for T2DM-S and HC.

Index	Tracts	T2DM-S	HC	Statistic	*p*-Value	Cohen’s d
1	TR_L	0.458 ± 0.05	0.457 ± 0.04	0.106	0.916	0.03
2	TR_R	0.470 ± 0.04	0.473 ± 0.47	0.240	0.811	0.07
3	CST_L	0.631 ± 0.06	0.632 ± 0.04	0.072	0.942	0.02
4	CST_R	0.622 ± 0.05	0.523 ± 0.14	3.820	<0.01**	0.98
5	CC_L	0.448 ± 0.05	0.40 ± 0.09	2.320	0.024	0.63
6	CC_R	0.418 ± 0.05	0.414 ± 0.07	0.213	0.832	0.06
7	Forceps major	0.573 ± 0.06	0.574 ± 0.05	0.060	0.952	0.02
8	Forceps minor	0.509 ± 0.04	0.516 ± 0.03	0.683	0.498	0.21
9	IFOF_L	0.435 ± 0.04	0.443 ± 0.02	0.784	0.437	0.24
10	IFOF_R	0.432 ± 0.03	0.438 ± 0.03	0.597	0.553	0.19
11	ILF_L	0.407 ± 0.03	0.293 ± 0.14	4.060	<0.01**	1.07
12	ILF_R	0.392 ± 0.04	0.397 ± 0.02	0.435	0.665	0.14
13	SLF_L	0.432 ± 0.04	0.418 ± 0.04	0.826	0.414	0.27
14	SLF_R	0.392 ± 0.04	0.288 ± 0.12	4.600	<0.01**	1.19
15	UF_L	0.486 ± 0.03	0.496 ± 0.04	0.981	0.332	0.30
16	UF_R	0.460 ± 0.04	0.473 ± 0.04	1.090	0.282	0.34
17	Arcuate_L	0.482 ± 0.04	0.421 ± 0.10	3.070	0.003	0.792
18	Arcuate_R	0.454 ± 0.04	0.403 ± 0.09	2.790	0.007	0.726

*Mean ± standard deviation (SD) is reported.*

**p < 0.01.

*T2DM-S, type 2 diabetes mellitus with subjective memory complaints; HC, health control; AD, Alzheimer’s disease; MD, mean diffusivity; TR, thalamic radiation; CST, corticospinal tract; CC, cingulum cingulate; IFOF, inferior fronto-occipital fasciculus; ILF, inferior longitudinal fasciculus; SLF, superior longitudinal fasciculus; UF, uncinate fasciculus; _R, right; _L, left; FA, fractional anisotropy.*

**TABLE 3:** Diffusion measures statistics along the fiber bundles.

	Tracts	T2DM-S	HC	Segments	Statistic	*p*-Values (correct)
FA	CST_R	0.68 ± 0.06	0.61 ± 0.17	82–84	2.5901	0.0053*
	SLF_R	0.40 ± 0.09	0.46 ± 0.06	94–100	2.5818	0.0048*
	Arcuate_L	0.39 ± 0.17	0.40 ± 0.04	44–61, 90–92	2.8014	0.0030*
	Arcuate_R	0.31 ± 0.16	0.40 ± 0.13	66–69	2.8157	0.0030*
MD	CST_L	0.83 ± 0.08	0.79 ± 0.03	23–27	2.6307	0.0059*
	CC_L	0.61 ± 0.27	0.75 ± 0.07	57–59, 71–80, 88–89	2.8525	0.0031*
	ILF_R	0.80 ± 0.15	0.66 ± 0.32	1–8, 69–70, 85	2.7307	0.0044*
	IFOF_L	0.93 ± 0.11	0.83 ± 0.06	1–11	2.7519	0.0039*
	IFOF_R	0.88 ± 0.18	0.74 ± 0.28	21–23	2.8899	0.0025*
AD	CST_L	1.53 ± 0.10	1.45 ± 0.05	13–29	1.8946	0.0443*
	Arcuate_L	0.95 ± 0.41	1.17 ± 0.05	46–56, 87–93	1.9673	0.0291*
RD	TR_L	0.72 ± 0.10	0.65 ± 0.06	1–8	2.6378	0.0054*

*Mean ± standard deviation (SD) of difference nodes is reported.*

*p < 0.05.

*The table shows two sample t-test was carried out on automatic fiber quantification (AFQ) bundle profiles of subjects to get significant group differences along the tract. Along-the-tract diffusion value was corrected for multiple comparisons (p < 0.05, FWE correction). The p-value at a specific segment implies how much significance there is between patients and healthy controls for that particular bundle.*

### Model Performance

**FIGURE 2:**
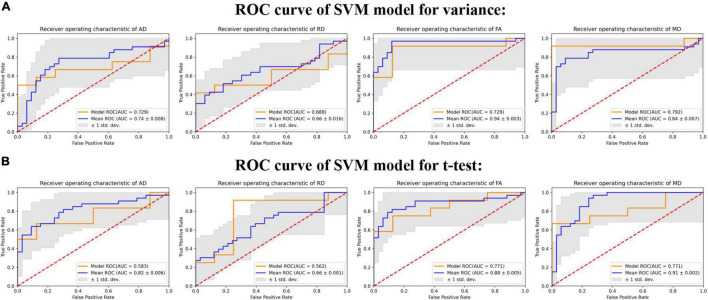
The ROC curves of different DTI-derived indexes in support vector machine (SVM) model. The figure depicts the ROC curve of the trained model (yellow curve) and the average ROC curve (blue curve) of the model after leave-one-out cross-validation (LOOCV), and the shaded part represents the standard deviation (SD) of area under the curve (AUC). **(A)** Shows the results of training the model with four DTI parameters after feature selection by variance filtering method (variance threshold = 1). **(B)** Shows the results of training the model with four DTI parameters after feature selection by two sample *t*-test (*p* < 0.05).

Given the relatively small sample size, we employ distinct DTI parameters as data to synthesize the differences of different fiber tracts. When we use the results of the two-sample *t*-test to screen features (shown as Figure 2B), FA-trained SVM model utilized 39 final point features and achieved a receiver operating characteristic AUC of 88% and an accuracy of 84.85% (*p* < 0.01). MD-trained SVM model utilized 49 final point features and achieved AUC of 91% and an accuracy of 75.76% (*p* < 0.01). RD-trained SVM model utilized eight final point features and achieved AUC of 66% and an accuracy of 60.61% (*p* = 0.07). AD-trained SVM model utilized 35 final point features and achieved AUC of 82% and an accuracy of 74.24% (*p* < 0.01). When we use the results of variance to select features (shown as Figure 2A), FA-trained SVM model utilized 192 final point features and achieved a receiver operating characteristic AUC of 94% and an accuracy of 87.88% (*p* < 0.01). MD-trained SVM model utilized 235 final point features and achieved AUC of 84% and an accuracy of 80.30% (*p* < 0.01). RD-trained SVM model utilized 57 final point features and achieved AUC of 66% and an accuracy of 63.64% (*p* = 0.042). AD-trained SVM model utilized 96 final point features and achieved AUC of 82% and an accuracy of 71.21% (*p* = 0.002). The detailed parameters are shown in Supplementary Tables S2, S3.

### Correlation With Clinical Features

**FIGURE 3:**
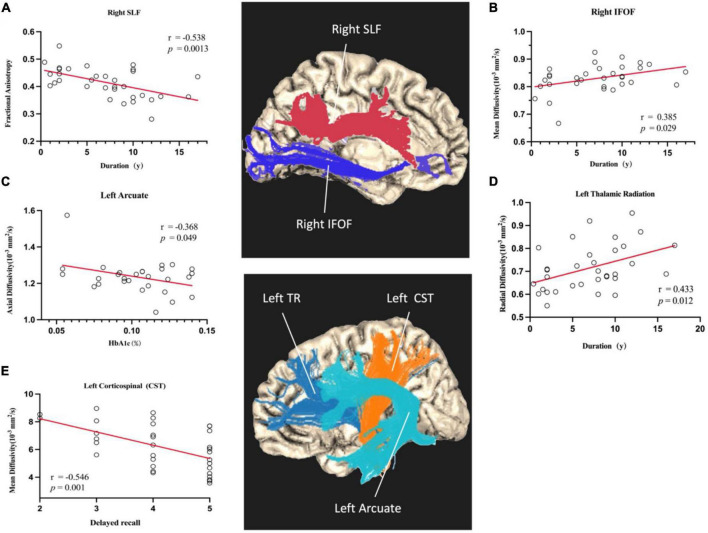
Scatterplots of partial correlation analyses between averaged DTI-derived indexes and HbA_1c_, delayed recall, and duration of disease. The red lines are the regression lines controlled for age, gender, and education. Correlation coefficients are noted as *r*, and *p* < 0.05 is considered as significant. **(A)** Shows the duration was negatively correlated with the mean FA of right SLF. **(B)** Shows the duration was positively correlated with the mean MD of right IFOF. **(C)** Shows the HbA_1c_ was negatively correlated with the mean AD of left Arcuate. **(D)** Shows the duration was positively correlated with the mean RD of left Thalamic Radiation. **(E)** Shows the delayed recall was negatively correlated with the mean MD of left CST.

Finally, clinical and cognition variables (duration of diabetes, delayed recall, and HbA_1c_) were correlated with diffusion characteristics as generated by AFQ along-the-tract analysis (*p* < 0.05). Lower FA in the right SLF tract (*r* = -0.538, *p* = 0.0013), higher RD in the TR tract (*r* = 0.433, *p* = 0.012), and higher MD in the IFOF tract (*r* = 0.385, *p* = 0.0029) were significantly associated with a long period of disease. Decreased AD in the left arcuate was associated with HbA_1c_ (*r* = −0.368, *p* = 0.049). In addition, we found a significant negative correlation between delayed recall and abnormal MD in the left corticospinal tract (*r* = −0.546, *p* = 0.001) (As shown in Figure 3).

## Discussion

In this AFQ-based study, we investigated the aberrant WM microstructure of T2DM and further investigated their correlation with SMC. The primary findings are as follows: First, our study indicates that the DTI parameters capture WM changes in T2DM patients before they are diagnosed with conventional neuropsychological test results. Second, the AFQ approach combined with machine learning algorithms can identify biomarkers related to the microstructure of WM in the early stage of T2DM. Third, the evaluation of the DTI parameters on T2DM patients demonstrated a correlation with the level of HbA_1c_, cognition performance, and the duration of disease. To the best of our knowledge, we are the first to assess the integrity of whole-brain WM microstructure in people with T2DM utilizing a recently established approach called AFQ. Our results may extend the precursor stage of T2DM related cognitive impairment to the SMC stage.

The identified fiber tracts are part in line with earlier reports for the injury pattern of WM integrity proposed in previous findings (Tan et al., 2016). WM injury may change along the trajectory of the bundle, but this change is not obvious in the entire fiber (Chandio et al., 2020; Chen et al., 2020). The destruction of WM in the brain is closely related to memory loss (Quan et al., 2020). Previous research has shown that the temporal and frontal lobes play an important role in diabetes-related cognitive impairment (Chen et al., 2014; Zhang et al., 2014). The relationship between the frontal and temporal lobes and language memory was also found in T2DM (Yau et al., 2009). This can be supported by the evidence that cerebral blood flow derived from glucose metabolism is observed in PET technology measurements (Quan et al., 2020). Although the underlying cognitive process remains unknown, the injury of these fiber tracts are thought to be related to the destruction of the internal network (Zhang et al., 2016). Evidence from relevant animal trials can support structural abnormalities in these fiber tracts and could lead to functional disconnections of brain networks (Biessels and Reagan, 2015). Therefore, it is speculated that the abnormality of the specific node of the WM fiber tracts is related to the aberration of the memory decline of T2DM patients. These results provide new insights into our understanding of neuropsychological mechanisms in these patients.

The T2DM-S group had significantly reduced FA and increased AD, RD, and MD on the WM tracts of both hemispheres, which might reflect earlier pathological changes, such as inflammation, microglial activation/accumulation, and impairment of WM integrity of cellular structures (Quan et al., 2020). FA can usually be used as an indicator of demyelination and axon integrity. However, models from animal studies have shown that increased RD reflects demyelination, while decreased AD suggests abnormalities in the axonal structure itself (Song et al., 2005; Hubner et al., 2017). In our study, the abnormal increase in AD in the left CST and arcuate, and RD in some nodes of the left TR indicates the explanation of myelination and the extensive abnormality of axon soma, which may prove the above statements. There were abnormalities within tracts connecting frontal and temporal lobes to other brain regions, which is already known through functional neuroimaging or gray matter volumetric studies to be abnormal in cognitive dysfunction (Yang et al., 2020). These bundles are mainly connected to the relevant brain areas of the default brain network (DMN, including the cingulum, SLF, and ILF), which are consistent with the results of previous studies (Tan et al., 2016; Sanjari Moghaddam et al., 2019). In a recent review, patients with T2DM show less functional connectivity compared with controls and reduced integrity of the WM in the default mode network (Wang et al., 2020). Abnormal right thalamus radiation was also found, which is the main pathway connecting the frontal lobe and thalamus. The IFOF is a major long-range tract with functional connectivity of distinct cortical brain regions associated with executive function, social cognition, attention, and semantic processing of language (Quan et al., 2020). Given the putative roles of ILF and IFOF in vision and language processing (Quan et al., 2020), our findings are compatible with the concept that WM injury can affect early memory complaints. As a result, the injury appears to be extensive, and it affects the left or right hemisphere. Multiple abnormal destruction of WM may indicate that diabetes-related risk factors can make the WM structure fragile (Ma et al., 2018).

These findings highlight the potential adverse effects of impaired glucose metabolism on cognition and brain structure. Although the loss of WM integrity is thought to be a possible early biomarker of neurodegeneration, little is known about the clinical implications of this result. The abnormal microstructure of diabetes-related cognitive impairment is related to pathological disorders of endocrine profile (Sanjari Moghaddam et al., 2019), including glucose toxicity, inflammation, oxidative stress, insulin resistance, blood-brain barrier destruction, and cerebral macro- and microvascular disease (Viazzi et al., 2017), leading to the disintegration and destruction of the myelin sheath and causing changes in cognitive function (Strachan et al., 2011). This association signifies that these defects may be caused by the diabetic impact on WM. For instance, disruption of WM integrity has been associated with slowing of processing speed and poor executive function (Vernooij et al., 2009). Furthermore, follow-up studies have shown that diabetes is associated with accelerated progression of brain atrophy, accompanied by a decline in processing speed and executive function (Falvey et al., 2013). The abovementioned fiber bundles may underlie attention and memory deficits and executive dysfunction. We found that the fiber bundles related to the duration of disease were concentrated in the left TR, the right SLF bundle, and the right IFOF bundle. As a transit station connecting various brain regions, the thalamus plays an important role in the Papez circuit through the structural connection of the medial temporal lobe (Xia et al., 2020), participates in memory, learning, information processing, and the integration of cross neural circuits, and plays an important role in the formation of cognitive function (Van der Werf et al., 2003). The abnormality of RD value of left TR is similar to many AD-related studies, and the connection between medial temporal cortex and thalamus is damaged (Damoiseaux et al., 2009; Delli Pizzi et al., 2014). With the extension of the duration of the disease, the degree of injury further increased, linked to the abnormalities in the right SLF and IFOF tracts, indicating that the long-term metabolic abnormalities have long-term and lasting changes in brain cognitive-related structures. Previous studies found that chronic hyperglycemia and oxidative stress play a key role in diabetes-related cognitive decline (Kielstein, 2013; Biessels et al., 2014; Strachan and Price, 2014; Rosness et al., 2016; Young-Hyman et al., 2016). The metabolic syndrome linked to diabetes may accelerate the disruption of the pathway of the brain, resulting in cognitive impairment (Zilliox et al., 2016). This study also found a correlation between AD and HbA_1c_ in the left arcuate bundle. In addition, we found evidence of a negative correlation between HbA_1c_ and WM microstructure (left arcuate) in patients with T2DM-S. Studies have shown that metabolic serum marker HbA_1c_ is associated with impaired cognitive function and WM integrity in healthy people (Repple et al., 2021). In the past, most DTI studies on people with MCI or AD have linked the WM damage score to global cognitive measures such as the (MMSE; Huang and Auchus, 2007). We found no significant correlation between the overall score of MMSE and MoCA with fiber bundle injuries in our investigation. Interestingly, there was a negative connection between the delayed recall of MoCA and the MD value of the left corticospinal tract, indicating a correlation between T2DM WM injuries and SMC symptoms. The results confirmed the effect of the reported WM bundle disruption on cognitive performance and are consistent with previous studies (Goldstein et al., 2009).

The difference in FA values along with the fiber bundles can be used as a potential optimal imaging biomarker for evaluating early brain injury in T2DM-S. Previous studies (Cabeen et al., 2017) have shown that the results of traditional voxel-based methods are affected by smoothing kernels. The AFQ method generates a mean streamline per subject with 100 equidistant points (Chandio et al., 2020), so it can provide more information about WM injury. The AFQ method can automatically identify the main intracranial fiber tracts and their abnormalities. We also found that there were abnormalities of RD and AD in some other tracts not detected by FA, including other tracts connecting the frontal lobe and temporal lobe with other brain structures (such as left TR, left CC, and left arcuate). This suggests that the FA measurement, which is considered to be the most sensitive to axonal structural integrity, seems to be more sensitive than other parameters in the early abnormal microstructure of T2DM-S. In the framework of SVM, we used a variety of DTI parameters to train the model, which show good performance. In our sample, compared with other parameters, the FA-based learning model shows the best accuracy (more than 80%) no matter what feature selection method was used. The two feature selection methods have little effect on the end results of the machine learning algorithm, indicating that the location of WM damage is fixed and special, which can be identified by machine learning methods. The above indicates that FA value is very sensitive to the extensive changes in the microstructure of T2DM-S.

We should recognize several limitations of the current study. First, although we used a CSD method to eliminate the influence of cross fibers on the results, some fiber bundles still have poor values (mainly some fine fibers), which may be a limitation of the DTI model. Second, the current study is limited by its relatively small sample size, and the severity of SMC is not quantified by a specific scale. As a result, our findings should be interpreted with caution and verified using a larger sample size. Third, we did not incorporate normal cognition T2DM and T2DM with MCI into our research, which will be one of our future study goals. Fourth, this study is an exploratory study to find the relationship between abnormal brain microstructure and SMC in patients with T2DM, so as to provide ideas for future research. Fifth, the cross-sectional nature of this study cannot demonstrate whether SMC causes WM abnormalities or whether WM differences were preexisting with T2DM. Longitudinal research will be used to solve these questions in the future (Yuan et al., 2018). Finally, only a general evaluation of cognitive impairment was carried out. Future research is needed to study the connection between WM integrity and other cognitive processes in T2DM-S by including more extensive measures of cognitive function (e.g., digit-span test and Stroop test).

## Conclusion

Biomarkers based on abnormal indicators on DTI segments can identify early T2DM patients with SMC. On the one hand, it shows that T2DM-S patients have structural alterations in WM in the early stage, which may be the basis of SMC. On the other hand, it also shows that DTI is an effective tool to measure the subtle injuries of fiber tracts. As a supplement to diabetic brain injury, the WM damage hypothesis of T2DM has been extended to SMC; the memory complaints of T2DM patients also have the same corresponding pathological basis of structural damage. A further understanding of the role of fiber tracts and their integrity in the cognitive performance of diabetes is helpful for us to understand the neuropsychological symptoms observed in diseases and make us closer to preserving specific neuroanatomical units.

## Data Availability Statement

The raw data and analysis scripts supporting the conclusions of this article will be available by the corresponding author on reasonable request.

## Ethics Statement

The studies involving human participants were reviewed and approved by the Lanzhou University Second Hospital, and the procedures conformed to the tenets of the Declaration of Helsinki. Written informed consent was obtained from all participants for their participation in this study. Written informed consent was obtained from the individual(s) for the publication of any potentially identifiable images or data included in this article.

## Author Contributions

JZ was responsible for the project administration. JW wrote and edited the original draft. GL and WB handled the experimental data collection. LM, GL, PZ, and WH conceived and designed the experiments. KA edited the manuscript with substantial input from JW. All authors critically revised the manuscript for important intellectual content.

## Conflict of Interest

KA was employed by the company Philips Healthcare. The remaining authors declare that the research was conducted in the absence of any commercial or financial relationships that could be construed as a potential conflict of interest.

## Publisher’s Note

All claims expressed in this article are solely those of the authors and do not necessarily represent those of their affiliated organizations, or those of the publisher, the editors and the reviewers. Any product that may be evaluated in this article, or claim that may be made by its manufacturer, is not guaranteed or endorsed by the publisher.

## Footnotes

1
http://vistalab.stanford.edu/

2
https://github.com/jyeatman/AFQ

3
http://surfer.nmr.mgh.harvard.edu/

4
https://www.mrtrix.org/

5
https://yeatmanlab.github.io/AFQ-Browser

6
https://seaborn.pydata.org/

7
http://white.stanford.edu/software

## Abbreviations

AFQ: automated fiber quantification
WM: white matter
FA: fractional anisotropy
MD: mean diffusivity
AD: axial diffusivity
RD: radial diffusivity
TR: thalamic radiation
CST: corticospinal
IFOF: inferior fronto-occipital fasciculus
ILF: inferior longitudinal fasciculus
SLF: superior longitudinal fasciculus
MoCA: montreal cognitive assessment
MMSE: mini-mental state examination.
