# Supplementary material for: Tractography in Type 2 Diabetes Mellitus With Subjective Memory Complaints: A Diffusion Tensor Imaging Study
Source: Front Neurosci. 2022 Apr 6;15:800420. doi: 10.3389/fnins.2021.800420 (PMC9019711; doi:10.3389/fnins.2021.800420)
Supplement: Supplementary file 1 [file Table_1.docx]

Supplementary Material

Table S1. Additional demographic information and clinical characteristics of T2DM patients

| **T2DM** | 2h-G  (mmol/l) | CHO  (mmol/L) | TG  (mmol/L) | HDL  (mmol/L) | C-p  (ng/ml) | INS  (pmol/ml) | use-ins |
| --- | --- | --- | --- | --- | --- | --- | --- |
| **Mean(SD)** | 16.7 (5.27) | 4.51 (1.08) | 2.64 (1.61 | 1.01 (0.21) | 1.09 (0.69) | 7.57 (5.32) | 16:17 |

Abbreviation: 2h-G, 2-h fasting blood glucose; CHO, cholesterol; TG, triglyceride; HDL, high density lipoprotein; C-p, C- peptide; INS, Fasting insulin; use-ins, use insulin.
