# Supplementary material for: Tractography in Type 2 Diabetes Mellitus With Subjective Memory Complaints: A Diffusion Tensor Imaging Study
Source: Front Neurosci. 2022 Apr 6;15:800420. doi: 10.3389/fnins.2021.800420 (PMC9019711; doi:10.3389/fnins.2021.800420)
Supplement: Supplementary file 2 [file Table_2.docx]

Supplementary Material

**Table S2**．SVM model performance of values for t-tests

| **T-tests** | ***Accuracy*** | ***Sensitivity*** | ***Specificity*** | ***F1-score*** | ***Mean AUC*** | ***p-Value*** |
| --- | --- | --- | --- | --- | --- | --- |
| FA | 84.85% | 81.82% | 87.88% | 84.38% | 88±0.5% | 0.001^*^ |
| MD | 75.76% | 69.70% | 81.82% | 74.19% | 91±0.2% | 0.0001^*^ |
| RD | 60.61% | 45.45% | 75.76% | 53.57% | 66±0.1% | 0.07 |
| AD | 74.24% | 75.76% | 72.73% | 74.63% | 82±0.6% | 0.0001^*^ |

Mean ± standard deviation (SD) of AUC is reported.

^*^*p* < 0.01.

Table S2 show various indicators of the evaluation model, including accuracy, sensitivity, specificity and F1 score, as well as the average AUC value after cross validation, and the *p* value generated after comparison with the random model (*p* < 0.01).
