# Supplementary material for: Tractography in Type 2 Diabetes Mellitus With Subjective Memory Complaints: A Diffusion Tensor Imaging Study
Source: Front Neurosci. 2022 Apr 6;15:800420. doi: 10.3389/fnins.2021.800420 (PMC9019711; doi:10.3389/fnins.2021.800420)
Supplement: Supplementary file 3 [file Table_3.docx]

Supplementary Material

**Table S3．**SVM model performance of values for variance

| **Variance** | ***Accuracy*** | ***Sensitivity*** | ***Specificity*** | ***F1-score*** | ***Mean AUC*** | ***p-Value*** |
| --- | --- | --- | --- | --- | --- | --- |
| FA | 87.88% | 84.85% | 90.91% | 87.50% | 94±0.3% | 0.0001^*^ |
| MD | 80.30% | 63.64% | 96.97% | 76.36% | 84±0.7% | 0.0001^*^ |
| RD | 63.64% | 51.52% | 75.76% | 58.62% | 66±1.6% | 0.042 |
| AD | 71.21% | 57.58% | 84.85% | 66.67% | 82±0.6% | 0.002^*^ |

Mean ± standard deviation (SD) of AUC is reported.

^*^*p* < 0.01.

Table S3 show various indicators of the evaluation model, including accuracy, sensitivity, specificity and F1 score, as well as the average AUC value after cross validation, and the *p* value generated after comparison with the random model (*p* < 0.01).

Note: The linear kernel has just one parameter (*C*) that controls the trade‐off between having zero training errors and allowing misclassifications; this parameter was fixed at C = 1 for all cases (default value) in accordance with previous neuroimaging studies (Li et al., 2014).

**Reference:**

Li, F., Huang, X., Tang, W., Yang, Y., Li, B., Kemp, G., et al. (2014). Multivariate pattern analysis of DTI reveals differential white matter in individuals with obsessive-compulsive disorder. *Human brain mapping* 35(6)**,** 2643-2651. doi: 10.1002/hbm.22357.
